# Intraocular lens implantation for patients with coloboma of the iris

**DOI:** 10.3892/etm.2014.1615

**Published:** 2014-03-12

**Authors:** JUANJUAN LI, YAN LI, ZHULIN HU, LEI KONG

**Affiliations:** 1 Department of Ophthalmology, The Fourth Affiliated Hospital of Kunming Medical University, Kunming, Yunnan 650021, P.R. China; 2 Department of Ophthalmology, The First Affiliated Hospital of Kunming Medical University, Kunming, Yunnan 650021, P.R. China

**Keywords:** iris coloboma, crystalline lens, man-made, surgical operation

## Abstract

The aim of this study was to analyze the techniques for intraocular lens (IOL) implantation in patients with coloboma of the iris. A retrospective cohort study was used to analyze the degree of iris coloboma and the characteristics of the crystalline lens in 56 patients with iris coloboma. The patients with a lesser degree of coloboma of the iris and an intact lens capsule were treated by iris suture and IOL implantation into the posterior chamber. Patients with an iris coloboma confined to one quadrant, severe iris atrophy and significant lens capsule coloboma were treated with an annular suture at the edge of the pupil and IOL implantation into the anterior chamber. Patients with a greater degree of iris coloboma and an intact lens capsule were treated with an artificial iris and IOL implantation. The patients were followed up for between five months and five years after surgery. Data relating to vision, photophobia, IOL location, postoperative complications and treatment were also obtained at follow-up. The vision of the patients was improved to varying degrees following the surgery, with the exception of those with amblyopia or serious corneal scars. The photophobia of the patients had also improved. The patients’ levels of satisfaction and comfort were deemed to be satisfactory. Early postoperative complications included hyphema, increased intraocular pressure and uveitis. However, serious complications such as corneal decompensation and IOL dislocation were not observed. Various techniques for IOL implantation were selected based on the degree of iris and lens capsule coloboma; these techniques were capable of improving the vision and photophobia of the patients.

## Introduction

The iris has important physiological functions with regard to regulating the amount of light that enters the eyes, increasing focal depth and decreasing eyeball aberrations (1). Patients with apriority, traumatic or surgical aniridia, or serious iris coloboma exhibit several symptoms, including serious photophobia, monodiplopia, glare and unsatisfactory corrected eyesight (2). Due to congenital dysplasia, trauma, surgery, the integrity of the iris is damaged or missing and normal morphology and size of pupil are damaged, referred to as the ‘iris defect’. Furthermore, these patients also present with cataracts and crystalline lens dislocation (3,4). Various surgical techniques may be used to conduct IOL implantation when patients exhibit with iris coloboma and cataracts (5,6). In the present study, a review of patients treated with various surgical techniques based on different iris coloboma and crystalline lens anomalies was carried out, and the results of the various treatments are reported.

## Materials and methods

### General data

A total of 56 patients, comprising 38 males and 18 females, with ages ranging from 2.5 to 45.2 years (mean, 21.6±2.4 years), who were treated with IOL implants at The Fourth Affiliated Hospital of Kunming Medical University (Kunming, China) from January 2006 to January 2011 were included in this study. The numbers of patients who required monocular and binocular treatment were 49 and 7, respectively. In total, 49 patients presented with traumatic iris coloboma with cataracts or crystalline lens coloboma and seven presented with congenital iris coloboma and cataracts. The best corrected visual acuity was <0.1 in 33 cases, 0.1–0.4 in 21 cases and 0.5 in two cases. All patients experienced photophobia and glare to varying degrees. Examination of the eye by B-scan ultrasound (ODM2000; Shanghai Huan Hee Medical Devices Co. Ltd. Shanghai, China) and ophthalmoscopy (OMEGA500, HEINE, HEINE Optotechnik, Herrsching, Germany) was carried out to exclude diseases of the fundus oculi. Various surgical techniques were used based on the different degrees of iris coloboma, atrophy and lens capsule damage. This study was conducted according to the Declaration of Helsinki and was approved by the Ethics Committee of the First Affiliated Hospital of Kunming Medical University. Informed consent was obtained from all participants.

### Surgical techniques

In total, 11 patients presented with iris coloboma confined to one quadrant with an intact lens capsule, of which eight exhibited traumatic iris coloboma with cataracts and three had apriority iris coloboma with cataracts. These patients were treated with iris sutures, cataract extirpation and IOL implantation into the posterior chamber. The surgery used was continuous circular capsulorhexis, which required either aspiration or phacoemulsification extraction of the cataract and the implantation of a collapsible type of IOL into the posterior chamber. A 10-0 suture and a 1–2 needle were used to suture the pupil.

A further six patients presented with an iris coloboma confined within one quadrant, severe iris atrophy, and significant crystalline lens coloboma caused by trauma. These patients were treated with an annular suture at the pupil edge and IOL implantation into the anterior chamber. A clear corneal incision was performed and sodium hyaluronate (Shandong Bausch & Lomb Freda Pharmaceutical Co. Ltd. Jinan, China) was injected into the anterior chamber. The edge of the damaged iris was continuously sutured with a polypropylene line in order to rebuild the pupil. Subsequently, the IOL was implanted into the anterior chamber, and the location of the IOL was adjusted to a central position.

The study included two patients who presented with binocular congenital iris coloboma and congenital cataracts, eight with traumatic cataracts caused by traumatic debridement and suturing of the monocular cornea, and two with binocular congenital cataracts treated with optical iridectomy. These patients were each treated with an artificial iris and IOL implantation. The surgery for these patients required aspiration, extracapsular cataract extraction, or phacoemulsification cataract extraction. A capsular tension ring with an iris diaphragm was implanted into the lens capsule. A Morcher Type 96G Partial Aniridia Ring (Morcher GmbH, Stuttgart, Germany) was implanted into the eyes of patients with partial iris coloboma, and a section of the iris diaphragm was rotated to correspond with the section of the iris coloboma. If the patients had a complete iris coloboma, two Morcher Type 50C Aniridia Rings (Morcher GmbH) were implanted, and the corresponding sections were folded to constitute a complete iris diaphragm. The tensile ring and IOL location were adjusted following the insertion of a collapsible type of IOL.

In total, 27 patients with iris coloboma in more than two quadrants, severe lens capsule coloboma, or crystalline lens dislocation in more than one quadrant were treated by IOL with iris implantation with a foldable intraocular lens. An incision of ~10 mm was opened on the edge of the corneosclera. The cataract was removed, with aspiration as required, or the crystalline lens was excised and sodium hyaluronate was injected into the anterior chamber. The IOL with iris was implanted into the ciliary groove. The polypropylene line was removed from the anterior chamber at the ten o’clock position located 15 mm behind the edge of the corneosclera and also removed from the four o’clock position. The line was subsequently removed from the incision beside the edge of the corneosclera, cut off and fastened to the loop of the IOL with iris. A knot was tied at the end of the line and was covered by a conjunctival flap.

## Results

### Iris sutures and IOL implantation

**Figure 1:**
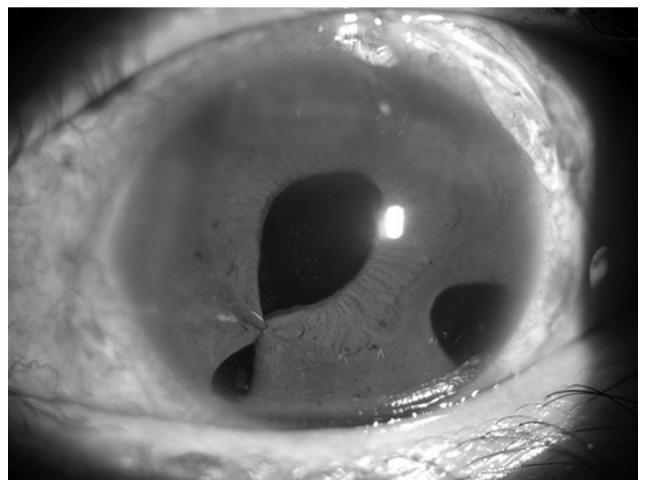
Anterior segment following iris suturing, cataract extirpation and intraocular lense (IOL) implantation into the posterior chamber. The pupil is oval in shape and the IOL is not shifted.

The vision of the eight patients with traumatic iris coloboma from the 11 treated with iris sutures, cataract extirpation and IOL implantation into the posterior chamber, significantly improved. Three of these eight patients had a corrected visual acuity of <0.1 and the other five patients had a best corrected visual acuity of between 0.1 and 0.5. Among the three patients with apriority iris coloboma, two patients had a corrected visual acuity of 0.5–0.8 and one patient had a corrected visual acuity of >0.8. The vision of these patients did not improve following surgery due to amblyopia. The patients did not exhibit photophobia. The patients’ pupils examined with a slit lamp were observed to be round or oval in shape. The diameter of pupils was between 3 and 4 mm, and the location of the posterior chamber IOL was not shifted (Fig. 1).

### Annular suture and IOL implantation

**Figure 2:**
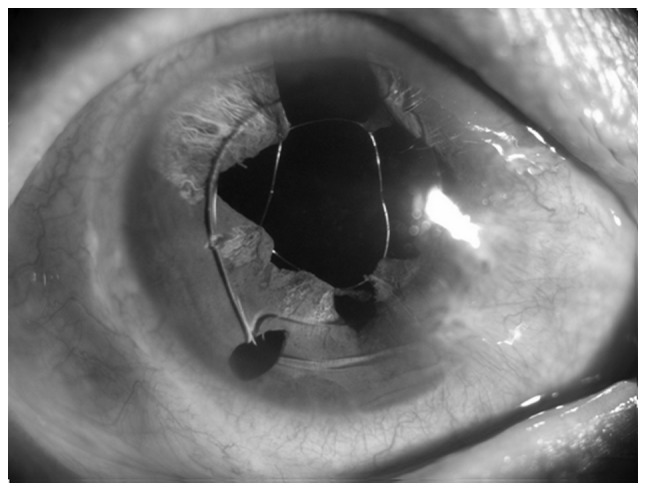
Anterior segment following annular suturing of the pupil edge and intraocular lense (IOL) implantation into the anterior chamber. A polypropylene line causes each section of the residual atrophic iris to form rounded pupils, and the reconstituted pupils are capable of supporting an IOL in the anterior chamber.

Of the six patients treated with an annular suture to the pupil edge and IOL implantation into the anterior chamber, the corrected visual acuity of four patients improved to 0.1–0.4, whereas the other two patients had a corrected visual acuity of 0.6. Furthermore, the patients no longer presented with photophobia. Under slit lamp observation, the polypropylene line caused each section of the residual atrophic iris to form rounded pupils; the reconstituted pupils were capable of supporting a IOL in the anterior chamber (Fig. 2).

### Artificial iris and IOL implantation

**Figure 3:**
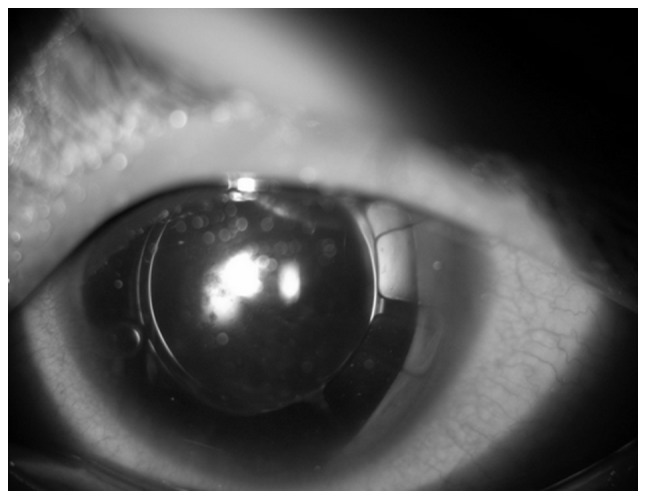
Anterior segment following the implantation of Morcher Type 50C artificial iris and intraocular lense (IOL). Two artificial irises form one round iris, and the IOL in the phacocele is not shifted.

Among the 12 patients treated with artificial iris and IOL implantation into the lens capsule, the corrected visual acuity of the two congenital cataract patients was <0.1 due to amblyopia. In the remaining 10 patients, the corrected visual acuity was 0.1–0.4 for six patients, 0.5–0.8 for three patients and >0.8 for one patient. There was no occurrence of photophobia. The artificial iris and IOL did not shift in the follow-up visit. The artificial iris constituted or complemented the coloboma iris, and the location of the IOL in the capsular bag did not shift (Fig. 3).

**Figure 4:**
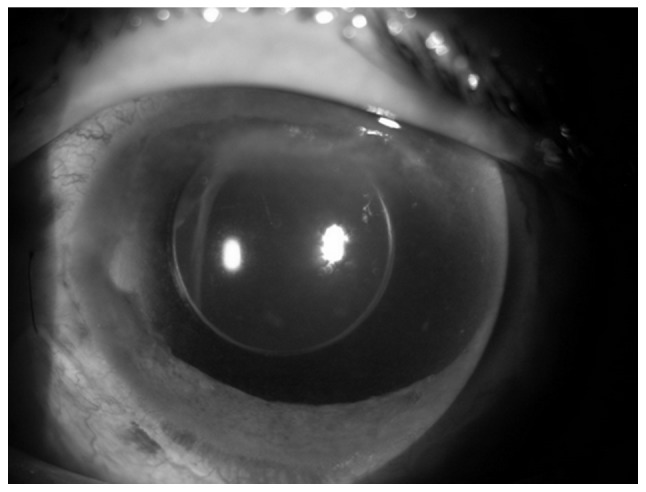
Anterior segment following intraocular lense (IOL) and iris implantation. The IOL with iris is located behind the remaining iris and is well placed in the center.

Of the 27 patients treated by IOL with iris implantation into the ciliary groove, the corrected visual acuity of five patients was <0.1. In addition, 14 patients had a corrected visual acuity of 0.1–0.4 and eight had a corrected visual acuity of 0.8. Of the 27 patients, 18 presented with no photophobia, and nine patients presented with slight photophobia. The IOL and optical parts shifted slightly in three patients. The IOL with iris was located in the ciliary sulcus and was well placed in the center. The patients’ photophobia improved to varying degrees following surgery (Fig. 4).

### Early postoperative complications

Early postoperative complications included hyphema, increased intraocular pressure and uveitis, which were improved following pharmacotherapy. The IOL and optical parts shifted slightly in three patients.

## Discussion

Researchers have attempted several different surgical techniques to solve the problems associated with IOL implantation and to improve the photophobic conditions of patients with iris coloboma. Previous studies have used a lid suture (7), corneal interlamellar dye (8) and colored corneal contact lenses (9) in order to solve photophobia following surgery. However, these methods have led to an unsatisfactory appearance with corneal discoloration and patient intolerance (7,10,11). To date, various surgical techniques and intraocular implantation methods have been developed.

When the extent of the iris coloboma is small, it is possible to directly suture the existing iris and reconstruct the pupils. In the present study, this surgical technique was applied to patients with an iris coloboma of small range, and whose remaining iris was capable of being sutured. This technique involves a simple surgery with no excessive pulling of the iris and minimizes the harm to the corneal endothelium and any reaction from the iris. The surgical techniques were chosen based on the condition of the phacocele (4). When the surplus iris was atrophic to varying degrees and the range of the iris coloboma was small, it was not possible to suture the iris to form a round pupil and decrease the patients’ photophobia. Thus, annular suturing of the iris using a polypropylene line was conducted. This technique may adequately utilize the remaining iris in order to reconstruct the pupils. It may also be a requirement for IOL implantation into the anterior chamber (12). For patients with a healthy phacocele, and whose crystalline lens was not shifted or had only a slight shift, IOL surgery with artificial iris implantation into the phacocele is the ideal technique for the treatment of iris coloboma with a cataracts. This technique fits in much the same position as normal physiology. Papillary block and a shift in the iris diaphragm or IOL rarely occurred. Furthermore, there was no friction with the ciliary body or remaining iris. Thus, the inflammatory response was low following surgery. Additionally this surgery is minimally invasive, and is achieved using a general clear corneal incision to the cataract (11). When the iris and phacocele were seriously damaged, the IOL technique, with the iris fixed in the ciliary groove, was used. Further postoperative complications were observed with this technique, such as intraocular hemorrhaging, inflammatory response and secondary glaucoma, due to the large size of the implant and brittle character (13,14).

Other than the previously mentioned methods, other surgical techniques have been used to solve the problems associated with IOL implantation in patients with iris coloboma, including the following: i) A prosthetic iris system, which may be capable of solving the aesthetic problems and photophobia associated with iris coloboma, as it is possible to make a personalized prosthetic iris that matches with the remaining iris with respect to position, size, and color (15). ii) An anterior chamber IOL with iris, in which an outer border of artificial iris acts as a holding device for the IOL positioned at the center of the implant. This IOL is implanted into the anterior chamber, and the holding device is fixed to the remaining iris (16). iii) An artificial iris and IOL with haptic parts, comprising a central IOL encircled by an artificial iris with a slender appendage used to fix the implant to the ciliary sulcus (17).

Following the rapid development of surgical techniques and intraocular implants, various methods have been made available for the treatment of patients with iris coloboma, wherein the implantation of an IOL is necessary. Once the range or degree of iris coloboma, the integrity of the phacocele, surgical skill, risk and cost have been estimated, the selection of various surgical techniques for solving the problems with the patients’ vision and photophobia is possible.
